# JC Virus Mediates Invasion and Migration in Colorectal Metastasis

**DOI:** 10.1371/journal.pone.0008146

**Published:** 2009-12-03

**Authors:** Alexander Link, Sung Kwan Shin, Takeshi Nagasaka, Francesc Balaguer, Minoru Koi, Barbara Jung, C. Richard Boland, Ajay Goel

**Affiliations:** 1 Division of Gastroenterology, Department of Internal Medicine, Charles A. Sammons Cancer Center and Baylor Research Institute, Baylor University Medical Center, Dallas, Texas, United States of America; 2 Department of Gastroenterological Surgery and Surgical Oncology, Okayama University Graduate School of Medicine Dentistry and Pharmaceutical Sciences, Okayama, Japan; 3 Division of Gastroenterology, Department of Medicine, University of California San Diego, La Jolla, California, United States of America; 4 Department of Gastroenterology, Hepatology and Infectious Diseases, Otto-von-Guericke University, Magdeburg, Germany; 5 Division of Gastroenterology, Department of Internal Medicine, Institute of Gastroenterology, Yonsei University College of Medicine, Seoul, Korea; 6 Department of Gastroenterology, Institut de Malalties Digestives i Metabòliques, Hospital Clínic, CIBEREHD, IDIBAPS, University of Barcelona, Barcelona, Catalonia, Spain; Saint Louis University School of Medicine, United States of America

## Abstract

**Introduction:**

JC Virus (JCV), a human polyomavirus, is frequently present in colorectal cancers (CRCs). JCV large T-Ag (T-Ag) expressed in approximately half of all CRC's, however, its functional role in CRC is poorly understood. We hypothesized that JCV T-Ag may mediate metastasis in CRC cells through increased migration and invasion.

**Material and Methods:**

CRC cell lines (HCT116 and SW837) were stably transfected with JCV early transcript sequences cloned into pCR3 or empty vectors. Migration and invasion assays were performed using Boyden chambers. Global gene expression analysis was performed to identify genetic targets and pathways altered by T-Ag expression. Microarray results were validated by qRT-PCR, protein expression analyses and immunohistochemistry. Matching primary CRCs and liver metastases from 33 patients were analyzed for T-Ag expression by immunohistochemistry.

**Results:**

T-Ag expressing cell lines showed 2 to 3-fold increase in migration and invasion compared to controls. JCV T-Ag expression resulted in differential expression of several genetic targets, including genes that mediate cell migration and invasion. Pathway analysis suggested a significant involvement of these genes with AKT and MAPK signaling. Treatment with selective PI3K/AKT and MAPK pathway inhibitors resulted in reduced migration and invasion. In support of our in-vitro results, immunohistochemical staining of the advanced stage tumors revealed frequent JCV T-Ag expression in metastatic primary tumors (92%) as well as in their matching liver metastasis (73%).

**Conclusion:**

These data suggest that JCV T-Ag expression in CRC associates with a metastatic phenotype, which may partly be mediated through the AKT/MAPK signaling pathway. Frequent expression of JCV T-Ag in CRC liver metastasis provides further clues supporting a mechanistic role for JCV as a possible mediator of cellular motility and invasion in CRC.

## Introduction

Colorectal cancer (CRC), with ∼150,000 new cases per year, is the third most frequent malignant disease, and with ∼50,000 deaths, constitutes the second leading cause of cancer mortality among men and women in the United States [1]. Mortality in CRC is usually caused by metastatic disease. Despite increasing efforts to diagnose CRC at an early stage with screening programs, more than 25% of patients are still diagnosed with metastatic disease, and an additional 25% eventually develop metastases. Unfortunately, the molecular mechanisms underlying the development of metastasis are poorly understood [2].

The assumption that viruses may be involved into the multistep process of carcinogenesis has a long history, and it is well accepted that 15–20% of cancers can be linked to chronic viral infections [3]. Most of the evidence comes from human papillomavirus and its role in cervical cancer, hepatitis B virus in hepatocellular carcinoma, and Epstein-Barr virus in lymphoproliferative diseases, Burkitt's lymphoma and nasopharyngeal carcinoma [3], [4]. Although there is convincing data to suggest a carcinogenic role for polyomaviruses in animal models, their role in the causation of human cancer is controversial in spite of accumulating evidence from various experimental studies [5], [6]. In the present study, we have investigated the role of human JC polyomavirus (JCV), which is known to cause progressive multifocal leucoencephalopathy and has also been frequently found in multiple gastrointestinal cancers including CRC, implying an oncogenic function in humans [7], [8].

JCV is a 5.13 kb, nonenveloped, double stranded, and closed circular DNA virus, which encodes 3 viral capsid proteins (VP1, VP2 and VP3), an agnoprotein, small (t-Ag), and large (T-Ag) transforming antigens. JCV T-Ag has a considerable sequence homology with T-Ag's of BKV and SV40. T-Ag is a multifunctional oncoprotein, which has the ability to bind and break DNA, and has helicase and ATPase activities [8]. Additionally, through direct protein-protein interaction, it can inactivate the key tumor suppressor proteins p53 and pRb, deregulating the cell cycle checkpoints and avoiding p53-mediated pro-apoptotic activity [8]. T-Ag may control cellular proliferation by deregulating the Wnt-signaling pathway through stabilization of β-catenin [9], [10]. Moreover T-Ag interacts with the IGF-IR signaling system, which contributes to cell transformation [11].

More than 30 years ago the carcinogenic potential of JCV was suggested in a hamster model [12], [13]. In recent years, several studies have been performed to evaluate the role of JCV in humans. The seroprevalence for the JCV viral capsid protein-1 in the adult population has historically been shown to be about 60–90%, although recent studies provide new evidence that the prevalence of JCV may be somewhat lower than that reported previously [14], [15]. Since JCV Mad-1 DNA can be found throughout the gastrointestinal tract the expression of T-Ag protein is believed to be a more suitable method for understanding oncogenic role of JCV in human cancers [16]. Although few groups have failed to detect JCV T-Ag expression in tumors, the majority of studies have provided increasing evidence for the expression of JCV T-Ag in CRC [17]. A recent study from our laboratory demonstrated that ∼50% of patients with CRC express T-Ag protein, and that T-Ag expression was exclusively present only in neoplastic colonic cells, but was never expressed in normal colonic mucosa [18], [19]. Further, in one of our earliest experiments we reported that JCV can induce chromosomal instability (CIN) in CRC cells [20]. Analysis of different tumor subtypes showed a clear association between JCV T-Ag expression and both CIN and the CpG island methylator phenotype in CRCs [19].

Recently, studies have shown that different DNA viruses may participate in the regulation of tumor cell invasion and migration to induce a metastatic phenotype. For instance, the E2 oncogene encoded by HPV, and the LMP1 oncoprotein of EBV virus, enhance cellular invasion through up-regulation of matrix-metalloproteinase-9 (MMP-9) [21]. Also, HBx protein of HBV as well as LMP1 can down-regulate E-cadherin and promote cellular motility by reducing cellular adhesion [22].

In view of the data on human oncogenic viruses, we hypothesized that JCV oncogenic proteins, in particular T-Ag, might mediate migration and invasion and contribute to malignant behavior in CRC. To address this hypothesis, we established an *in vitro* CRC cell model where we generated stable clones of JCV T-Ag-expressing lines. We first determined migration and invasion ability of the T-Ag expressing cells. Next, we performed global gene expression profiling studies to identify specific genetic targets and molecular pathways that may regulate T-Ag-induced changes in cellular motility. Herein, we demonstrate that JCV T-Ag transfection in CRC cells is associated with increased malignant behavior that in part is mediated through PI3K/AKT and MAPK pathways. In support of these *in vitro* data we provide evidence that JCV T-Ag expression is frequently present in CRC liver metastases.

## Materials and Methods

### Cells Culture and Transfection

Two human CRC cell lines, HCT116 (microsatellite instability or MSI) and SW837 (microsatellite stable or MSS) were obtained from the American Type Culture Collection (ATCC, Manassas, VA). Cells were cultured in IMDM medium (Invitrogen, Rockville MD) supplemented with 10% fetal bovine serum with 5% CO_2_ at 37°C. Cell lines were transfected with full-length JCV-early transcript coding region cloned into a pCR3 vector (called JCV_E_, kindly provided by Dr. Richard Frisque, The Pennsylvania State University, University Park, PA) or an empty vector as transfection control. JCV_E_ coding region encodes all 5 transforming proteins including T-Ag, t-Ag and the three splice variants (T'165, T'136 and T'135) [23]–[25]. Transfection was performed with Effectene (QIAGEN, Valencia, CA) using standard protocol with some modifications. Briefly, 24 h post-transfection, cells were washed and thereafter grown in standard medium for another 48 h for transient transfection experiments. Alternatively, cells were plated into 6 well plates for stable transfection following selection with 600–1000 µg/ml of G418. Stable clones were maintained in 200 µg/ml G418. JCV T-Ag expression was regularly monitored by RT-PCR or Western immunoblotting. Wherever indicated, the cells were treated with the selective PI3K inhibitor LY294002 (Cell Signaling, MA) at a concentration of 25 µM or the selective MEK1/2 inhibitor- U0126 (Cell Signaling, MA) at a concentration of 10 µM. Both LY294002 and U0126 stocks were prepared at 10 mM concentration and stored at −20°C until used.

### Primary CRC and Liver Metastasis Specimens

To study the JCV T-Ag expression in both primary tumors and CRC metastasis, we obtained 33 pairs of matching sporadic metastatic primary CRCs tissues and corresponding liver metastasis specimens from the same patients collected consecutively at Okayama University Hospital, Okayama, Japan. All patients provided written informed consent, and the study was approved by institutional review boards. Clinical and demographical data of the patients are presented in **Table 1**.

**Table 1:** JCV T-Ag expression in primary colorectal cancers and liver metastasis in correlation with clinico-pathological characteristics.

Demographic data	Total n (%)	Primary CRC			Liver metastasis		
		T-Ag positive n(%)	T-Ag negative n(%)	p-value	T-Ag positive n(%)	T-Ag negative n(%)	p-value
**All cases**	33 (100)	31 (94)	2 (6)		24 (73)	9 (27)	
**Sex**							
Female	15 (45)	14 (93)	1 (7)	NS	11 (73)	4 (27)	NS
Male	18 (55)	17 (92)	1 (6)		13 (72)	5 (28)	
**Age (years)**							
Mean age ± SD	60.8±12.6	61.6±11.9	48.5±23.3	NS	63.7±11.2	52.8±13.5	**0.026**
<65	16 (48)	15 (94)	1 (6)	NS	10 (63)	6 (37)	NS
≥65	17 (52)	16 (94)	1 (6)		14 (82)	3 (18)	
**Localization**							
Proximal	10 (30)	9 (90)	1 (10)	NS	7 (70)	3 (30)	NS
Distal	23 (70)	22 (96)	1 (4)		17 (74)	6 (26)	
Cecum	1 (3)	-	-		-	-	
Ascending	6 (18)	-	-		-	-	
Transverse	3 (9)	-	-		-	-	
Descending	2 (6)	-	-		-	-	
Sigmoid	9 (27)	-	-		-	-	
Rectum	12 (36)	-	-		-	-	
**Differentiation**							
Moderate	27 (82)	26 (96)	1 (4)	NS	20 (74)	7 (26)	NS
Well diff. and mucinous	6 (18)	5 (83)	1 (17)		4 (67)	2 (33)	
**Lymph node metastasis**							
N0–N1	21 (64)	19 (91)	2 (9)	NS	15 (71)	6 (29)	NS
N2–N4	12 (36)	12 (100)	0 (0)		9 (75)	3 (25)	
N0	2 (6)	-	-		-	-	
N1	19 (58)	-	-		-	-	
N2	10 (30)	-	-		-	-	
N3	1 (3)	-	-		-	-	
N4	1 (3)	-	-		-	-	
**Stage at primary tumor operation**							
II–III (no liver metastasis)	10 (30)	9 (90)	1 (10)	NS	6 (60)	4 (40)	NS
IV (with liver metastasis)	23 (70)	22 (96)	1 (4)		18 (78)	5 (22)	
II	1 (3)	-	-		-	-	
III	9 (27)	-	-		-	-	
IV	21 (64)	-	-		-	-	
Local recurrence	2 (6)	-	-		-	-	
**Time to liver metastasis surgery**							
Mean months ± SD	9.1±12.5	8.4±11.5	20±28.3	NS	6.2±8.7	16.8±17.7	**0.027**
Stage II–III	19.3±12.0	-	-		14.8±9.6	26.0±13.3	NS
Stage IV	5.3±10.2	-	-		4.1±6.4	9.4±18.3	

NS: non-significant (p>0.05) using where appropriate Fishers exact test or unpaired t-test.

### Microarray Analysis

Microarray gene expression analyses were performed on JCV T-Ag expressing cells and control cell lines. Cells were harvested at 70–80% confluency. Total RNA was isolated using the RNeasy mini-kit (QIAGEN, Valencia, CA) and amplified using Illumina's TotalPrep RNA Amplification Kit. RNA integrity was assessed using the Agilent 2100 Bioanalyzer. Labeled cRNA was hybridized overnight to Human HT-12 V3 chips, washed, and scanned on an Illumina BeadStation-500. Illumina's BeadStudio version 3.1 was used to generate signal intensity values from the scans, subtract background, and scale each microarray to the median average intensity for all samples (per-chip normalization). The data were transferred to GeneSpring GX7.3 (Agilent Technologies, Santa Clara, CA) and after normalization of data from T-Ag transfected cells with vector control cell lines, unsupervised analysis were performed using the following criteria: a) transcripts must be detected in at least one sample (p<0.01)–PALO and b) must demonstrate at least 1.5-fold up- or down-regulated expression from the median intensity in at least one sample–1.5xUDALO. Ingenuity pathway analysis (IPA) (Ingenuity Systems, Inc. CA, USA) was performed to analyze and categorize differentially expressed genes into different functional pathways. To validate the reproducibility of these data we performed semi-quantitative and quantitative Power SYBRgreen® PCR amplifications from 2 independent passages of vector (V) and T-Ag transfected cells prior to analyzing gene expression results.

### Real Time and Reverse Transcription PCR

For reverse transcription-PCR (RT-PCR), total RNA was extracted from frozen cell pellets with the RNeasy mini-kit (QIAGEN, Valencia, CA). RNA was reverse transcribed to cDNA from 1 µg of total RNA using random hexamers and Advantage RT-for PCR Kit (Clontech Laboratories, CA). For the determination of JCV expression in the transfected cell lines, we used the previously published JEX primers [26] and TaqMan customized primers that specifically detect JCV T-Ag mRNA sequences.Primer sequences and condition are listed in the **Table S1**.

### Western Immunoblotting

Pellets from T-Ag expressing and control cells were lysed in buffer containing 0.5 M Tris pH 6.8, 80% glycerol, 1.2 g sodium dodecyl sulfate and 0.5 M EDTA. Heat-denaturated cell lysates were subjected to electrophoresis on 10% denaturing polyacrylamide gels and proteins transferred to PVDF membranes (Amersham Hybond™-P, GE Healthcare). The blots were probed with appropriate dilutions of primary and secondary antibodies (horseradish peroxidase-conjugated IgG). Signal detection was performed following exposure of the membranes to the ECL reagent (Amersham™, GE Healthcare) and final visualization using the Storm imaging system. Following primary antibodies were used for western immunoblotting: mouse monoclonal antibody cocktail against JCV T-Ag (clones PAb2003, PAb2023, PAb20024, PAb2030, PAb2001, PAb2000 kindly provided by Dr. R. Frisque [27], [28]) at 1∶50 dilution; rabbit antibodies (Cell Signaling, MA) at a dilution range of 1∶500 to 1∶1000: phospho-AKT (Ser473), AKT, phospho-p44/42 MAPK (Thr202/Tyr204), p44/42 MAPK; mouse anti-β-actin antibody (Clone AC-15; at dilution of 1∶32000). Secondary goat anti-mouse (sc-2005) and goat anti-rabbit (sc-2004) antibodies were used at a dilution of 1∶5000 with 1 h incubation (Santa Cruz, CA).

### Migration and Invasion Assays

*In vitro* migration and invasion assays were performed using Boyden chambers (BD Biosciences, Bedford, MA) with 8-µm-pore size membranes with Matrigel (for invasion assays) or without matrigel (for migration assays). Briefly, 5 or 10×10^4^ cells were suspended in 500 µl of serum-free medium in the absence or presence of various selective inhibitors. Complete medium containing FBS was added to the bottom wells of the plate. Thereafter the plates were incubated for 72 h at 37°C in 5% CO2. After incubation, non-migrated cells were removed from the upper surface of the chamber with a cotton swab. Migrated/invaded cells were stained either with Diff-Quick™ stain or DAPI for nuclear DNA in an immunofluorescence assay. The images from at least 4 representative fields of each membrane were quantitatively measured with ImageJ software (NIH, USA). Each experiment was repeated at least 3 times to confirm the reproducibility of data.

### Cell Proliferation Assays

Proliferations analysis were performed with calorimetric Cell Proliferation ELISA, BrdU kit (Roche Diagnostics, Indianapolis, IN). Briefly, equal amount of the cells were cultured in 96-well plates. After 72 h, BrdU was added to the wells for labeling of proliferating cells. After 4 h incubation the cells were fixed and peroxidase-conjugated anti-BrdU antibody was added to the cells. BrDU incorporation in proliferating cells was quantitatively determined using a standard microplate reader.

### Immunofluorescence

Cells were grown on glass cover slips placed inside 24-well plates. Cells were washed with PBS, fixed with 4% formaldehyde, permeabilized with 0.1% Triton X-100 for 5 min, and blocked with 10% goat serum (Zymed, San Francisco, CA) for 30 min. The cells were subsequently incubated with appropriate dilutions of primary antibodies overnight, followed by 1 h incubations with fluorescein-conjugated secondary antibodies (Alexa Fluor® 594 goat anti-mouse IgG (Invitrogen, OR)). Finally, the cover slips were sealed with ProLong® Gold with DAPI (Invitrogen, OR) and visualized using an AxioSkop2 multichannel epifluorescence microscope and images acquired using AxioVision software (CarlZeiss Inc. Thornwood, NY).

### Immunohistochemistry

Staining for JCV T-Ag was performed on 4 µm paraffin-embedded CRC and metastasis tissue sections as described previously[18], [19]. Slides were incubated overnight with primary mouse monoclonal antibody against SV40 T-Ag, which cross reacts with JCV T-Ag (clone PAb416, 1∶40 dilution, Calbiochem, CA) followed by incubation in Dako EnVision™ labeled polymer (Dako Sytometion Inc., Carpinteria, CA). Staining was developed by reaction with diaminobenzide (DAB) and counterstained with hematoxylin. JCV-inoculated hamster brain tumor tissue (kindly provided by Dr. Kamel Khalili, Temple University, Philadelphia, PA) was used as a positive control for JCV-specific staining. Brown chromogen nuclear complexes indicated T-Ag expression, which was evaluated by 2 independent examiners (AL and AG) who were blinded to the clinical data.

### Statistical Analysis

All data were analyzed using SPSS 11.5 (Chicago, IL, USA) and Graph Pad Prism 4.0 (San Diego, CA, USA) statistical software. The differences between two groups were analyzed using Student's t-tests or where appropriate Fisher's exact test. Differences between more than two groups were analyzed using repeated measures ANOVA and Bonferroni's multiple comparisons as a *post hoc* test. Two sided p-values of <0.05 were regarded significant.

## Results

### JCV T-Ag Was Expressed at Both mRNA and Protein Levels in Transfected Cells

**Figure 1:**
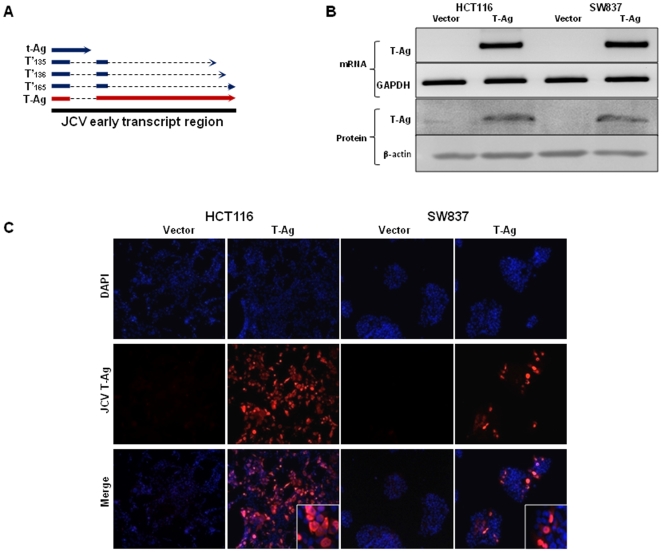
JCV T-Ag expression in transfected colorectal cancer cells. (A) An illustration of the JCV early transcript region that codes for 5 early transforming proteins, T-Ag, t-Ag and the 3 splice variants T'_165_, T'_136_, T'_135_. T-Ag is predominant protein expressed after transfection (marked in red). (B) RT-PCR and Western immunoblotting gel images depicting JCV T-Ag specific mRNA and protein expression in stably transfected cells (indicated as T-Ag), while no T-Ag expression was observed in control cell lines (V, vector transfected). GAPDH (RT-PCR) and β-actin (WB) were used as loading controls. (C) Immunofluorescence staining with JCV T-Ag antibody shows nuclear expression of JCV T-Ag in transfected HCT116 and SW837 cells lines. The images were taken at a final magnification of 630×.

JCV T-Ag expression is present in approximately 50% of CRC's, but its expression in colon cancer cell lines has not been described previously. At the outset of this study, we analyzed a subset of CRC cell lines and none of the studied cell lines showed presence of either JCV T-Ag mRNA or protein expression (data not shown). To establish an *in vitro* model for JCV T-Ag expression, we transfected a JCV T-Ag carrying plasmid into two prototypic colon cancer cell lines; HCT116, which is a microsatellite unstable cell line and is representative of Lynch syndrome colorectal cancers due to an underlying germline mutation in the DNA mismatch repair gene, *MLH1*; and SW837 cells, which are microsatellite stable but harbor mutations in the *p53* tumor suppressor gene (**Figure 1A**). Both cell lines were permissive for stable transfection, and maintained T-Ag mRNA and protein expression after >50 passages as indicated by RT-PCR and immunoblotting (**Figure 1B**). Since JCV early transcript region allows expression of all 5 transforming proteins including T-Ag, t-Ag and the three splice variants of large T-Ag (T'165, T'136 and T'135), we performed western immunoblotting with a cocktail of specific JCV antibodies to determine the expression of specific early proteins that are expressed in JCV_E_ transfected colon cancer cell lines. We found that transfection of JCV_E_ plasmid was predominantly associated with the expression of JCV T-Ag, suggesting that the other early proteins may not play a major role in this in-vitro cell culture system in contrast to their potential role in JCV-induced lytic infection in humans (**Figure S1**). Following transfection, we failed to detect any morphological changes in cellular morphology of JCV_E_ and vector transfected cells. Immunofluorescence staining with JCV T-Ag specific antibody further complemented RT-PCR and Western blotting data, in which T-Ag expression was predominantly nuclear in both transfected cell lines (**Figure 1C**).

### JCV Transfection Resulted in Increased Invasion and Migration

**Figure 2:**
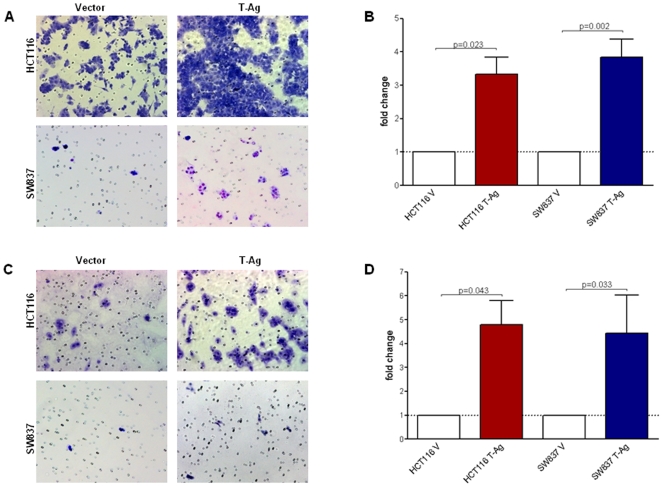
JCV T-Ag increases migration and invasion *in vitro*. Migration and invasion assays were performed in Boyden chambers without and with Matrigel respectively. Representative images from one of the three independent experiments showing migration (A) and invasion (B) in control and T-Ag transfected HCT116 and SW837 cell lines (200× magnification). The bar graphs in panels B and D are quantitative determinations of data obtained using ImageJ cell counter software from 3 independent experiments. As indicated, T-Ag transfection (red and blue bars) showed significantly increased migration and invasion (*p<0.05*) in both cell lines.

To test the hypothesis that one of the mechanisms for JCV T-Ag-mediated carcinogenesis may be through increased cellular invasion and migration, we first performed *in vitro* invasion and migration assays in T-Ag- and vector-transfected cell lines. As demonstrated in **Figure 2**, we observed a significant increase in both migration (**Figures 2A & 2B**) and invasion (**Figures 2C & 2D**) in HCT116 and SW837 cells following T-Ag transfection. Although there were slight variations between different cell passages used in independent experiments, T-Ag expressing cells persistently demonstrated increased migration in both HCT116 (mean±SE, 3.33±0.51 fold, *p = 0.023*) and SW837 cells (3.84±0.54 fold, *p = 0.02*) when the results were normalized to vector transfected control cells. We also observed a pronounced increase in invasion in HCT116 (4.78±1.03 fold, *p = 0.043*) and SW837 cells (4.63±1.45 fold, *p = 0.033*) following T-Ag transfection. Since changes in malignant phenotype may partly relate to changes in proliferation, we performed proliferation assay and found no difference in proliferation between JCV T-Ag expressing and vector transfected control HCT116 cells, and a small increase in proliferation in SW837 cells (data not shown).

### JCV Transfection Modulated Expression of Several Metastasis Associated Genes in AKT and MAPK Pathways

**Figure 3:**
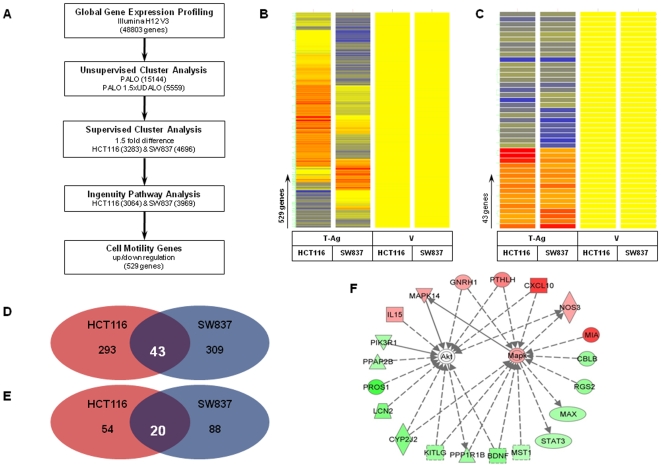
Global gene expression analysis of JCV T-Ag transfected cells. (A) The flow chart represents the strategy used for gene expression analysis. Using unsupervised analysis we selected 5559 genes. Dendograms in panels B and C illustrate clustering analysis of JCV-T-Ag transfected cells compared to vector controls. Gene trees are represented on the horizontal axis, while condition trees are represented on the vertical axis. The color conventions for all maps are as follows: red indicates over-expressed transcripts, blue are under-expressed transcripts, and yellow indicates transcripts that did not deviate from the controls. Clustering analysis in panel B illustrates Ingenuity pathway analysis which revealed 529 genes that are involved in regulation of cell motility. Of these, a subset of 43 genes that are specifically involved in migration or invasion and were up- or down-regulated in both cell lines are shown in panels C (as dendogram) and D (Venn diagram). Of this group of 43 genes, 20 genes that are directly or indirectly involved in AKT and MAPK pathways were shared between both HCT116 and SW837 cells as shown in panel E (Venn diagram) and panel F (as a schematic interaction of individual genes with each other). Red indicates up-regulated and green indicates down-regulated genes following T-Ag transfection (panel F).

Next, in order to further evaluate potential genetic targets and molecular pathways that might mediate increased migration and invasion in T-Ag expressing CRC cell lines, we performed global gene expression profiling in T-Ag transfected and vector transfected cell lines. In order to narrow down a subset of plausible genes that might mediate cellular migration and invasion, we performed unsupervised clustering analysis and selected a group of 5559 genes from a total of 48803 genes that were more than 1.5 fold significantly up- or down-regulated in at least one of the cell lines as described in the Methods (**Figure 3A**). Next, using Ingenuity pathway analysis software, we discovered that 529 genes that are involved with cellular motility were differentially expressed in T-Ag expressing HCT116 and SW837 cells. Although the two analyzed cell lines are representative of very different colon cancer subtypes, further analysis revealed that 43 of 529 differentially expressed genes were commonly up- and down-regulated in both HCT116 and SW837 cell lines (**Figure 3B, 3C & 3D**). These data indicated that very likely, this subset of genes might plausibly be responsible for increased migration and invasion following T-Ag transfection. Indeed, upon an independent data analyses, we identified 74 genes in HCT116, and 108 genes in SW837 cell line that directly or indirectly associated with AKT and MAPK signaling pathways (**Figure 3E**). From a total of 43 up- or down-regulated migration- and invasion-related genes from both cell lines, 20 genes specifically participated in AKT or MAPK signaling pathways (**Figure 3F**). Details on the microarray gene list are presented as supplementary data (**Table S2**). Based on this analysis, we surmised that alterations in AKT and/or MAPK signaling pathways may play an important role in phenotypic changes induced by JCV T-Ag transfection in CRC cells.

**Figure 4:**
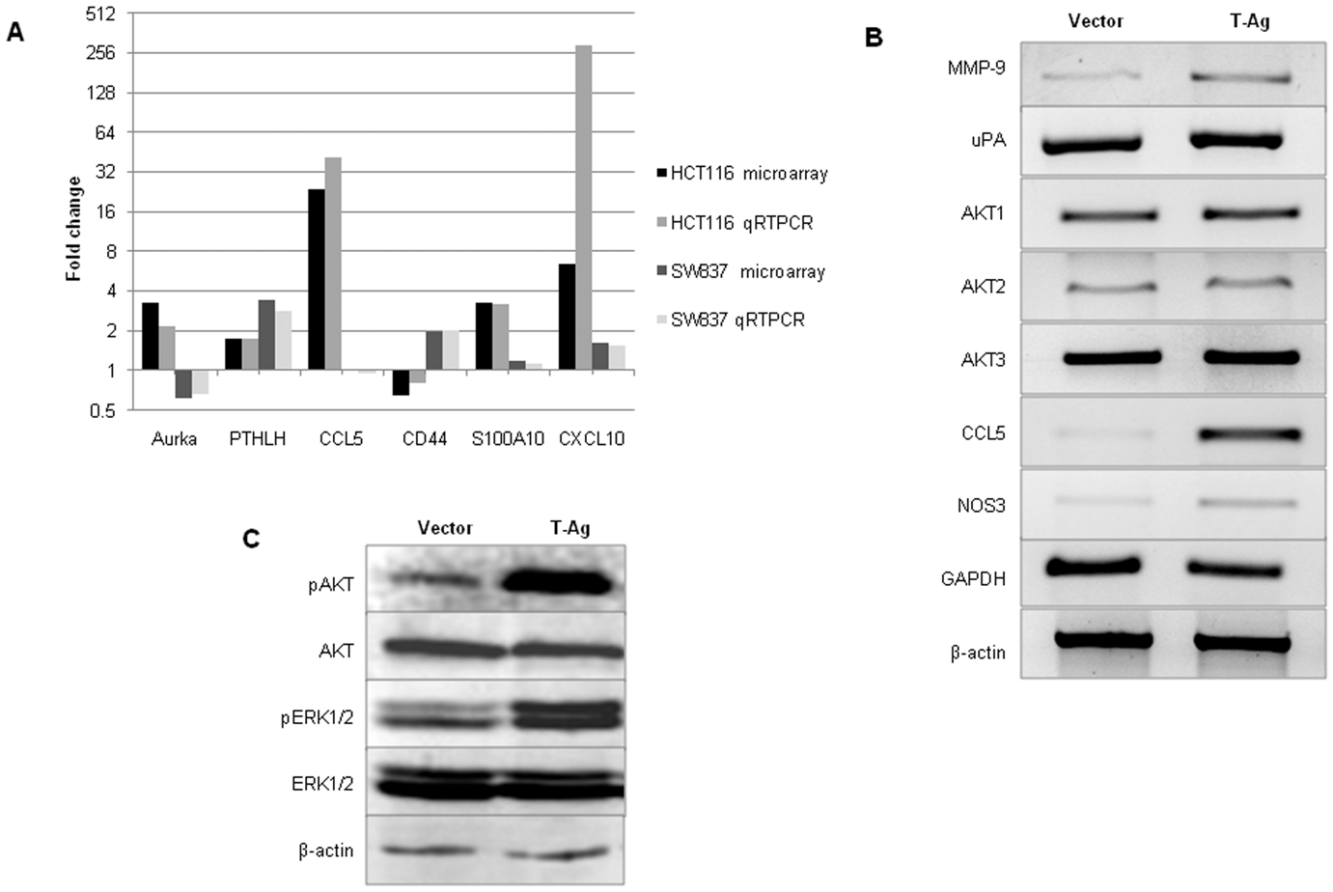
Gene expression changes following T-Ag transfection in HCT116 cells. (A) Microarray global gene expression data were validated in a randomly selected subset of genes by quantitative RT-PCR. Data are represented as the mean fold change normalized to vector cells. As indicated, there was a tight correlation between microarray and qRT-PCR in each instance. (B) A subset of cancer-metastasis related genes including MMP-9, uPA, CCL5 and NOS3 showed increased expression following T-Ag transfection in HCT116 cells. (C) Western blotting data indicated increased phosphorylation of AKT as well as ERK1/2 in T-Ag transfected cells in comparison to control cell lines.

In order to validate microarray gene expression data, we performed qRT-PCR analysis on a randomly selected subset of genes that showed significant changes in gene expression in at least one of the cell lines. As shown in **Figure 4A**, despite slight differences in absolute fold-changes in gene expression results between microarray data and qRT-PCR results, we observed a strong correlation between both methods.

### JCV Transfection Caused Activation of AKT and MAPK Pathways in Colonic Cells

We next questioned whether T-Ag transfection in CRC cells is associated with activation of either the AKT or MAPK pathways. Since MSI CRCs are inherently less metastatic, we performed these experiments with HCT116 cells, which are MSI-positive and demonstrated increased migration and invasion following T-Ag transfection in Boyden chamber assays. First we studied the changes in gene and protein expression of selected genetic candidates that are associated with increased migration, invasion or metastasis in CRC. As shown in the **Figure 4B**, we observed significant up-regulation in the mRNA transcripts of MMP9, CCL5, and NOS3 in T-Ag versus vector transfected HCT116 cells. Only a modest increase in uPA, and no change in AKT mRNA expression, were observed between vector and T-Ag transfected HCT116 cells. We then asked whether JCV T-Ag transfection might lead to activation of AKT and ERK1 and ERK2. Western immunoblotting revealed increased expression of phosphorylated AKT (Ser473) and both phosphorylated ERK1 and ERK2 (Tyr 202 and Tyr 204) in T-Ag expressing cells compared to vector carrying HCT116 cells (**Figure 4C**). In concordance with the gene expression data, we found no changes in total AKT or ERK1/2 protein expression.

### AKT/MAPK Pathway Inhibitors Blocked Migration and Invasion in JCV T-Ag Expressing Cells

**Figure 5:**
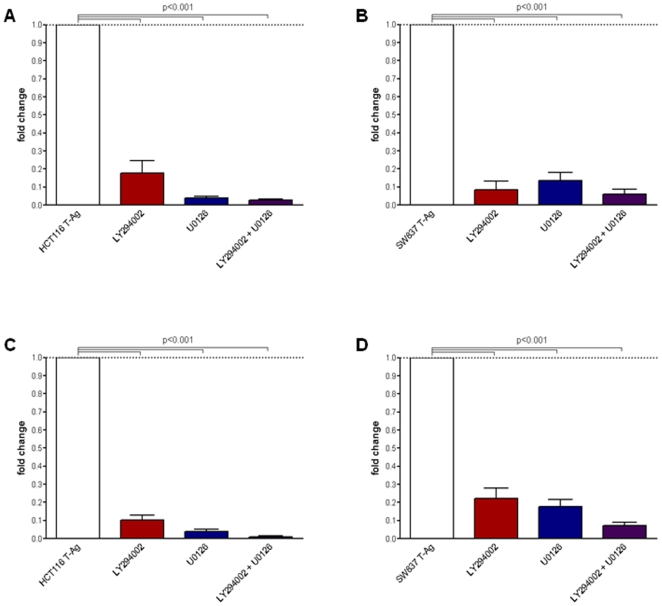
PI3K and MAPK pathway inhibitors reduced migration and invasion of JCV T-Ag transfected cells. Migration (panels A and B) and invasion (panels C and D) were performed using Boyden chambers without or with Matrigel. LY294002 (25 µM) and U0126 (10 µM) were added to both upper and lower chambers. Cells that invaded through Matrigel, and migrated through the 8 µm pores in the membrane were stained with DAPI. Cell counting was performed with ImageJ software. Data in bar graphs demonstrate fold changes normalized to vector cells (means±SE) obtained from 3 independent experiments. Significant inhibition in both migration and invasion was observed in both HCT116 and SW837 cells with LY294002 and U0126 individually and in combination.

We reasoned that if increased migration and invasion in T-Ag expressing cells was due to the activation of PI3K and MAPK pathways, specific PI3K/AKT and MAPK pathway inhibitors should be able to inhibit the migration and invasion potential in these cells. For these experiments, T-Ag expressing cell lines were treated with LY294002 and U0126 singly or in combination, followed by *in vitro* invasion and migration analyses using Boyden chambers. As expected, both inhibitors strongly reduced phosphorylation of AKT and ERK1/2 when used individually, however, the effects were additive when were used in combination (data not shown). As shown in **Figure 5**, LY294002 and U0126 significantly reduced migration (**Figures 5A and B**; *p<0.001*, T-Ag transfected versus both inhibitors individually or in combination) and invasion (*p<0.001*; **Figures 5C and D**) in HCT116 and SW837 cells. These data strongly support the concept that JCV T-Ag may induce migration and invasion, and is regulated through the PI3K and MAPK pathways.

### JCV T-Ag Is Frequently Expressed in Liver Metastasis

To further substantiate our *in vitro* results, we tested whether expression of JCV T-Ag might be associated with an increased risk for metastasis. For these studies, we examined JCV T-Ag expression in 33 patient using matching pairs of primary sporadic CRCs and the corresponding liver metastasis. Clinical characteristics of the patients are presented in **Table 1**. The majority of the samples obtained for this study were from patients with distal CRCs, mostly moderately differentiated, and 70% had stage IV tumors at the time of the primary tumor resection. Mean follow up time from primary diagnoses to liver metastasis surgeries was 9.1±12.5 months.

**Figure 6:**
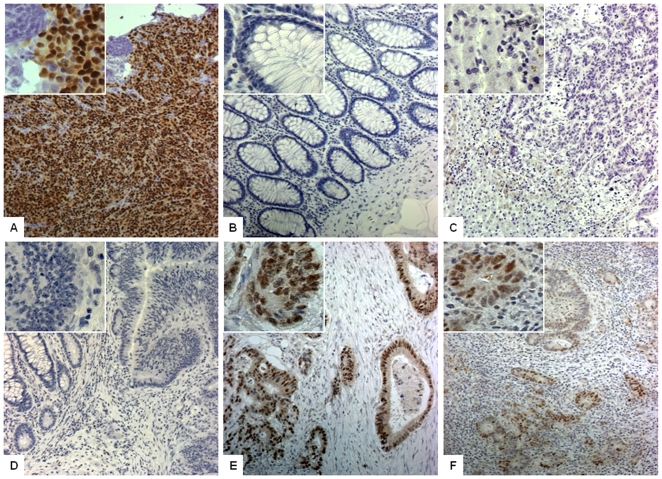
JCV T-Ag expression in primary CRC tumors and in liver metastasis. Staining was performed with Pab416 antibody against SV40 T-Ag with cross reactivity to JCV T-Ag. Images were obtained at 100× and 400× magnification (as shown in the insets within each photomicrograph) (A) Positive control staining from hamster brain tissue infected with JC virus shows strong nuclear T-Ag expression. Normal colonic mucosa (B) and liver hepatocytes (C) showed no JCV T-Ag expression and were used as negative controls with each staining. (D) A representative example of a CRC and colonic mucosa with no T-Ag expression. (E) A photomicrograph indicating strong nuclear T-Ag expression in invasive primary colonic tumor (F) The image illustrates T-Ag-specific expression in liver metastasis in cancer cells but no expression in surrounding normal liver hepatocytes.

Representative examples of JCV T-Ag positive and negative staining are shown in **Figure 6**. Normal colonic mucosa (**Figure 6B**) as well as liver cells (**Figure 6C**) showed no T-Ag staining in comparison to the positive control tissue (**Figure 6A**). Unexpectedly, although variable staining intensity was observed, T-Ag expression was present in 31 (93.9%) primary CRCs (**Figure 6D & 6E** **;** **Table 1**). Next, upon analysis of T-Ag expression in liver metastases from these same patients, we found that 24 (72.7%) of the patients showed JCV T-Ag expression also in the liver metastasis (**Figure 6F**). In 6 cases (19%) there was no T-Ag expression in the liver metastasis, although T-Ag expression was present in the primary tumor. None of the cases showed T-Ag positive expression in the liver metastasis if the primary tumor did not express this oncogene. Subgroup analysis revealed that JCV T-Ag expression in the liver metastasis was more frequently present in higher tumor stages and older age, supporting the hypothesis that JCV expression might be associated with an advanced stage of disease. Additionally, T-Ag expression in liver metastasis was associated with a shorter time interval between primary tumor resection and liver metastasis surgery in patients with stage II–III disease.

## Discussion

This work suggests that JCV infection is capable of mediating some of the most virulent aspects of tumor behavior in CRC. Transfection of JCV early transcript region into CRC cells and subsequent expression of JCV T-Ag induces phenotypic changes manifested through an increased potential for migration and invasion. These phenotypic alterations were further associated with the increased transcription of multiple genes that are likely to be involved in metastatic behavior, specifically, through the AKT and MAPK pathways. We also demonstrated that JCV T-Ag expression is not only present in primary CRCs, but is frequently present in liver metastases. Additionally, we found a correlation between JCV T-Ag expression in liver metastasis and more advanced tumor stage, lending support to our *in vitro* results, and suggesting a novel mechanism for the role of T-Ag in the progression of CRC.

In spite of the evidence suggesting the involvement of JCV in CRC more than a decade ago [29], the role of JCV in human cancer is currently poorly understood. An oncogenic role for JCV has been demonstrated in several animal studies including rodents and primates (reviewed in [30]), but the lack of an adequate *in vitro* model has hampered our understanding of the role of JCV in human cancer.

Notwithstanding that JCV may have a multitude of effects on tumor cells, in this study we focused our investigation on understanding JCV-induced changes in the migration and invasion of tumor cells. In this study, we intentionally selected CRC cell lines from both MSS and MSI genetic backgrounds because T-Ag expression has been shown to correlate with both categories of CRC [19]. We observed that JCV-transfected HCT116 and SW837 cells gained an increased ability for migration and invasion. In-vitro Matrigel based methods to study invasion and migration have been used for decades in laboratory-based research, and it has been shown that these assays correlate well with the in-vivo metastatic behavior of the cells [31], [32]. Our data corroborate some of the previous work done on other oncogenic DNA viruses like HPV, HBV and EBV, where changes in the modulation of cell adherence, motility, invasion and interaction with the microenvironment are common features [33]. One can argue that despite the structural and genomic differences between the DNA viruses, oncogenic viruses target similar tumor suppressor proteins (i.e., p53 and pRB) to orchestrate their activity. Along similar lines, there has been a suggestion that the PI3K/AKT or MAPK signaling pathways may mediate cellular migration and invasion by oncogenic viruses [34], as we observed in this study.

Although the role of JCV in malignant behavior has attracted little attention in the past, in an early experiment, JCV Mad-1 strain induced highly malignant, metastatic neuroblastomas in hamsters [35]. In another study, Khalili's group demonstrated that subcutaneous transplantation of T-Ag expressing cells, but not T-Ag negative cells, resulted in the development of massive tumors in experimental animals [36].

After obtaining evidence for increased cellular invasion and migration upon the introduction of JCV T-Ag in colon cancer cells, we were able to demonstrate that these phenotypic changes were associated with marked alterations in the expression of a large number of genes. These results are in line with a previous study by Nerurkar and colleagues in which the authors have analyzed gene expression alterations following transfection of a full-length JCV plasmid into glial cells [37]. Although we observed overlapping changes in gene expression results between our study and this previous report, we also noticed other differences including reduced activation of interferon related genes. Our observations for differences in gene expression profiles between HCT116 and SW837 cells following JCV_E_ transfection highlights the contribution of genetic (mutations, deletions etc) and epigenetic (methylation of gene promoters) factors that might mediate JCV-induced gene expression alterations in humans. We observed that various genes *CD44, CCL5, VEGFA, DSCR1, AURKA, RUNX2* and *MMP9* were significantly altered following JCV T-Ag expression, and prior data supports the role of these genes in migration and invasion behaviors in CRCs [38]. These observations further argue against the solitary regulation of individual genes by T-Ag, and rather support a broader role for this oncogene in mediating the expression of multiple targets that cumulatively impact the metastatic phenotype in CRC.

Ingenuity pathway analysis of data from differentially expressed genes in T-Ag transfected cells identified a subset of genes that are associated with cellular migration and invasion through the AKT and MAPK signaling pathways. It was recently shown that activation of ERK1/2 is a predictor of a poor prognosis in CRC, and strongly associates with *K-RAS* mutations [39]. AKT expression has also been associated with progression in colon carcinogenesis, but it has not been shown to be associated with prognosis [40]. However, key mechanistic role has been shown for AKT2 in CRC metastasis [41]. In this study, we could demonstrate that transfection of JCV T-Ag into the MSI-positive HCT116 cell line results in increased expression of both phosphorylated AKT and ERK1/2 proteins, supporting the interpretation that JCV T-Ag expression supports an AKT-mediated metastatic phenotype in CRC cells.

Several recent studies have highlighted the importance of AKT and MAPK pathways in JCV biology. For instance, JCV entry into the cell requires tyrosine kinase activity and JCV T-Ag activates the MAPK pathway through phosphorylation of ERK1/2 [42]. In medulloblastoma cell lines, prolonged activation of AKT positively correlates with the presence of JCV T-Ag expression [11]. From another perspective, increases in TGFβ as well as ERK expression have been shown to be involved in JCV multiplication, an effect that can be reversed upon treatment with PD98059 or U0126 [43]. We made similar observations as treatment with PI3K/AKT and MAPK pathway inhibitors strongly inhibited JCV T-Ag-associated migration and invasion, implicating the involvement of these pathways. Although we were able to identify the involvement of the AKP/MAPK pathway in JCV-mediated motility in colon cancer cells, our study did not address the specific roles of individual signaling molecules in these pathways. One of the unique features of our study is that we transfected complete JCV early transcript coding region, which associates with the expression of all 5 potential transforming including novel T-Ag splice variants previously shown by Frisque's group [24]. In this study, we predominantly observed expression of JCV T-Ag only in both transfected cell lines. Although we cannot exclude with certainty the role of t-Ag and other T'- proteins, based upon our results it is reasonable to speculate that T-Ag may be the primary mediator of a metastatic phenotype in colon cancer cells, and some of these effects may directly or indirectly be influenced by the activation of AKT/MAPK pathways [44], [45]. Nonetheless, future studies are required to better appreciate the functional consequences of changes in expression of MAPK/AKT pathway genes, and to determine whether these alterations are a direct manifestation of T-Ag transfection.

To evaluate possible clinical relevance of our *in vitro* data we also examined T-Ag expression in 33 sporadic CRC tumors and the associated liver metastases. Surprisingly, more than 90% of the tumors expressed JCV T-Ag, which is greater than what has been previously described in sporadic CRCs [19]. We acknowledge the possibility that one of the reasons for a higher frequency of T-Ag expression in this collection of tumors might due to selection bias of the tumors from known metastatic CRCs, compared to the unselected collections in previous publications. In addition, we did not have access to clinical data for the chemotherapy regimens prior to surgery, which could potentially select for JCV T-Ag expressing cells. Due to the limited availability of the clinical tissue specimens, we were unable to evaluate AKT and ERK1 expression in the primary CRCs and liver metastases tissues - future studies are required to address these questions. Nonetheless, we believe that we have analyzed a reasonable collection of primary tumors and liver metastases and for the first time we show not only that JCV T-Ag is expressed in primary CRC tumors, but also is frequently found in their liver metastases at a much higher frequency than what have been previously shown for non-metastatic sporadic CRCs [10], [19]. Although our data for T-Ag expression in liver metastases from patients with CRC will require independent validation in future prospective studies, currently, these results permit us to draw several conclusions. First, together with the *in vitro* data, these data indicate that JCV T-Ag expression in CRC may be associated with metastatic cellular behavior. Second, the presence of T-Ag expression in primary CRC and the liver metastases raises the possibility of integration of JCV into the cancer cell genome, as the origin of these cells is presumably from putative cancer stem cells.

In summary, this study suggests a novel role for JCV T-Ag in human CRC. We demonstrate that transfection of JCV early transcripts into cancer cells could increases migration and invasion through up-regulation of metastasis-associated genes. Increased expression of phosphorylated AKT and ERK1/2 and the reversible effect of PI3K/AKT and MAPK pathway inhibitors imply, at least in part, the involvement of PI3K/AKT and MAPK pathways. Detection of JCV T-Ag expression in primary CRCs and their metastases suggests a novel role for JCV T-Ag in metastasis during the late stages of CRC. Based on this data, we propose that JCV T-Ag may play a broader role than previously thought, and may be mechanistically involved in the late stages of these tumors.

## Supporting Information

Table S1 Primers and product lengths for semi-quantitative and TaqMan RT-PCR.
(0.05 MB DOC)

Table S2 List of differently expressed genes related to AKT or MAPK pathways based on Ingenuity pathway analysis (IPA).
(0.17 MB DOC)

Figure S1 Protein expression for T-Ag, t-Ag and T'-proteins in JCV_E_ transfected HCT116 and SW837 cell lines.
(1.72 MB TIF)
