# Supplementary material for: JC Virus Mediates Invasion and Migration in Colorectal Metastasis
Source: PLoS One. 2009 Dec 3;4(12):e8146. doi: 10.1371/journal.pone.0008146 (PMC2781631; doi:10.1371/journal.pone.0008146)
Supplement: Table S1 — Primers and product lengths for semi-quantitative and TaqMan RT-PCR. (0.05 MB DOC) [file pone.0008146.s001.doc]

**Table S1: Primers and product lengths for semi-quantitative and TaqMan RT-PCR**

| ***Gene name*** | ***Forward Primer*** | ***Reverse Primer*** | ***Product size (bp)*** |  |
| --- | --- | --- | --- | --- |
| *JCV-T-Ag (TaqMan)* | ACCAGGATTCCCATTCATCTGTTC | AGCCTGATTTTGGTACATGGAATAGTT | 69 |  |
| *JCV-T-Ag (JEX)* | TTCTACTAGTATGTATTCCACCAGGATTCC | CCTTCTCGAGTCTGCATGGGGGAACATTCC | 256 |  |
|  |  |  |  |  |
| *Akt1* | GCTGGACGATAGCTTGGA | GATGACAGATAGCTGGTG | 383 |  |
| *Akt2* | GGCCCCTGATCAGACTCTA | TCCTCAGTCGTGGAGGAGT | 276 |  |
| *Akt3* | GCAAGTGGACGAGAATAAGTCTC | ACAATGGTGGGCTCATGACTTCC | 329 |  |
| *AURKA* | GTCACAAGCCGGTTCAGAAT | AAGTCTTCCAAAGCCCACTG | 199 |  |
| *β-actin* | TACCACTGGCATCGTGATGGACTC | TCCTGCTTGCTGATCCACATCTGC | 642 |  |
| *β-actin (qPCR)* | GCCCTGAGGCACTCTTCCA | CGGATGTCCACGTCACACTTC | 100 |  |
| *CCL5* | CGCTGTCATCCTCATTGCTA | ACACACTTGGCGGTTCTTTC | 196 |  |
| *CD44* | CCCAGATGGAGAAAGCTCTG | TTCAGGTGGAGCTGAAGCAT | 188 |  |
| *CXCL10* | CCAATTTTGTCCACGTGTTG | GAGATCTTTTAGACCTTTCCTTGC | 141 |  |
| *GAPDH* | GAGGTGAAGGTCGGAGTC | GAAGATGGTGATGGGATTTC | 250 |  |
| *MMP9* | CAACATCACCTATTGGATCC | CGGGTGTAGAGTCTCTCGCT | 480 |  |
| *NOS3* | CGTGTGAAGAACTGGGAGGT | TGGAGCTGTAGTACTGGTTGATG | 205 |  |
| *PTHLP* | GGAGACTGGTTCAGCAGTGG | TGGATTTCTGCGATCAGATG | 196 |  |
| *S100A10* | AAATTCGCTGGGGATAAAGG | CCCGCAATTAGGGAAAAGA | 181 |  |
| *uPA* | CAGAGACACTAACGACTTCAGGG | GAGGATTGGATGAACTAGGCTAAA | 366 |  |
