# Supplementary material for: JC Virus Mediates Invasion and Migration in Colorectal Metastasis
Source: PLoS One. 2009 Dec 3;4(12):e8146. doi: 10.1371/journal.pone.0008146 (PMC2781631; doi:10.1371/journal.pone.0008146)
Supplement: Table S2 — List of differently expressed genes related to AKT or MAPK pathways based on Ingenuity pathway analysis (IPA). (0.17 MB DOC) [file pone.0008146.s002.doc]

**Table S2:** List of differently expressed genes related to AKT or MAPK pathways based on Ingenuity pathway analysis (IPA)

| **Symbol** | **Entrez Gene Name** | **Location** | **Family** |
| --- | --- | --- | --- |
| **HCT116 and SW837: up-regulated genes** | |  |  |
| CXCL10 | chemokine (C-X-C motif) ligand 10 | Extracellular Space | cytokine |
| IL15 | interleukin 15 | Extracellular Space | cytokine |
| NOS3 | nitric oxide synthase 3 (endothelial cell) | Cytoplasm | enzyme |
| MAPK14 | mitogen-activated protein kinase 14 | Cytoplasm | kinase |
| GNRH1 | gonadotropin-releasing hormone 1 | Extracellular Space | other |
| MIA | melanoma inhibitory activity | Extracellular Space | other |
| PTHLH | parathyroid hormone-like hormone | Extracellular Space | other |
|  |  |  |  |
| **HCT116 and SW837: down-regulated genes** | |  |  |
| CYP2J2 | cytochrome P450, family 2, subfamily J, polypeptide 2 | Cytoplasm | enzyme |
| BDNF | brain-derived neurotrophic factor | Extracellular Space | growth factor |
| KITLG | KIT ligand | Extracellular Space | growth factor |
| MST1 | macrophage stimulating 1 (hepatocyte growth factor-like) | Extracellular Space | growth factor |
| PIK3R1 | phosphoinositide-3-kinase, regulatory subunit 1 (alpha) | Cytoplasm | kinase |
| CBLB | Cas-Br-M ecotropic retroviral transforming sequence b | Nucleus | other |
| PROS1 | protein S (alpha) | Extracellular Space | other |
| RGS2 | regulator of G-protein signaling 2, 24kDa | Nucleus | other |
| PPAP2B | phosphatidic acid phosphatase type 2B | Plasma Membrane | phosphatase |
| PPP1R1B | protein phosphatase 1, regulatory (inhibitor) subunit 1B | Cytoplasm | phosphatase |
| MAX | MYC associated factor X | Nucleus | transcription regulator |
| STAT3 | signal transducer and activator of transcription 3 | Nucleus | transcription regulator |
| LCN2 | lipocalin 2 | Extracellular Space | transporter |
|  |  |  |  |
| **HCT116: up-regulated genes** | |  |  |
| CCL5 | chemokine (C-C motif) ligand 5 | Extracellular Space | cytokine |
| IL8 | interleukin 8 | Extracellular Space | cytokine |
| CDC42 | cell division cycle 42 (GTP binding protein, 25kDa) | Cytoplasm | enzyme |
| HPSE | heparanase | Plasma Membrane | enzyme |
| PLCE1 | phospholipase C, epsilon 1 | Cytoplasm | enzyme |
| PLCG1 | phospholipase C, gamma 1 | Cytoplasm | enzyme |
| CLEC11A | C-type lectin domain family 11, member A | Extracellular Space | growth factor |
| FGF2 | fibroblast growth factor 2 (basic) | Extracellular Space | growth factor |
| MDK | midkine (neurite growth-promoting factor 2) | Extracellular Space | growth factor |
| PDGFB | platelet-derived growth factor beta polypeptide | Extracellular Space | growth factor |
| CDKN1A | cyclin-dependent kinase inhibitor 1A (p21, Cip1) | Nucleus | kinase |
| ERBB3 | v-erb-b2 erythroblastic leukemia viral oncogene homolog 3 (avian) | Plasma Membrane | kinase |
| ILK | integrin-linked kinase | Plasma Membrane | kinase |
| MAP3K8 | mitogen-activated protein kinase kinase kinase 8 | Cytoplasm | kinase |
| MAPK11 | mitogen-activated protein kinase 11 | Cytoplasm | kinase |
| MAPK12 | mitogen-activated protein kinase 12 | Cytoplasm | kinase |
| MAPKAPK3 | mitogen-activated protein kinase-activated protein kinase 3 | Nucleus | kinase |
| PTK2B | PTK2B protein tyrosine kinase 2 beta | Cytoplasm | kinase |
| PTK6 | PTK6 protein tyrosine kinase 6 | Cytoplasm | kinase |
| CAV1 | caveolin 1, caveolae protein, 22kDa | Plasma Membrane | other |
| EDN1 | endothelin 1 | Extracellular Space | other |
| GAB2 | GRB2-associated binding protein 2 | Cytoplasm | other |
| HMGB1 | high-mobility group box 1 | Nucleus | other |
| HSP90AA2 | heat shock protein 90kDa alpha (cytosolic), class A member 2 | Unknown | other |
| HSP90AB1 | heat shock protein 90kDa alpha (cytosolic), class B member 1 | Cytoplasm | other |
| ICAM2 | intercellular adhesion molecule 2 | Plasma Membrane | other |
| IRS2 | insulin receptor substrate 2 | Cytoplasm | other |
| ITGA6 | integrin, alpha 6 | Plasma Membrane | other |
| ITGB5 | integrin, beta 5 | Plasma Membrane | other |
| MAP2 | microtubule-associated protein 2 | Cytoplasm | other |
| MARCKS | myristoylated alanine-rich protein kinase C substrate | Plasma Membrane | other |
| MUC1 | mucin 1, cell surface associated | Plasma Membrane | other |
| STMN1 | stathmin 1/oncoprotein 18 | Cytoplasm | other |
| WNT3A | wingless-type MMTV integration site family, member 3A | Extracellular Space | other |
| DUSP1 | dual specificity phosphatase 1 | Nucleus | phosphatase |
| PTPN6 | protein tyrosine phosphatase, non-receptor type 6 | Cytoplasm | phosphatase |
| CALR | calreticulin | Cytoplasm | transcription regulator |
| JUNB | jun B proto-oncogene | Nucleus | transcription regulator |
| RUNX2 | runt-related transcription factor 2 | Nucleus | transcription regulator |
|  |  |  |  |
| **HCT116: down-regulated genes** | |  |  |
| LIF | leukemia inhibitory factor (cholinergic differentiation factor) | Extracellular Space | cytokine |
| BTC | betacellulin | Extracellular Space | growth factor |
| GRP | gastrin-releasing peptide | Extracellular Space | growth factor |
| NRG1 | neuregulin 1 | Extracellular Space | growth factor |
| CSF1R | colony stimulating factor 1 receptor | Plasma Membrane | kinase |
| FYN | FYN oncogene related to SRC, FGR, YES | Plasma Membrane | kinase |
| RET | ret proto-oncogene | Plasma Membrane | kinase |
| CD44 | CD44 molecule (Indian blood group) | Plasma Membrane | other |
| EFNA1 | ephrin-A1 | Plasma Membrane | other |
| ERRFI1 | ERBB receptor feedback inhibitor 1 | Cytoplasm | other |
| SPRY4 | sprouty homolog 4 (Drosophila) | Plasma Membrane | other |
| PTPRH | protein tyrosine phosphatase, receptor type, H | Plasma Membrane | phosphatase |
| FOXC1 | forkhead box C1 | Nucleus | transcription regulator |
| MYC | v-myc myelocytomatosis viral oncogene homolog (avian) | Nucleus | transcription regulator |
|  |  |  |  |
| **SW837: up-regulated genes** | |  |  |
| CSF2 | colony stimulating factor 2 (granulocyte-macrophage) | Extracellular Space | cytokine |
| IL12A | interleukin 12A | Extracellular Space | cytokine |
| GNB2L1 | guanine nucleotide binding protein, beta polypeptide 2-like 1 | Cytoplasm | enzyme |
| ADORA2A | adenosine A2a receptor | Plasma Membrane | G-protein coupled receptor |
| CXCR4 | chemokine (C-X-C motif) receptor 4 | Plasma Membrane | G-protein coupled receptor |
| ANGPT1 | angiopoietin 1 | Extracellular Space | growth factor |
| CTGF | connective tissue growth factor | Extracellular Space | growth factor |
| NTF3 | neurotrophin 3 | Extracellular Space | growth factor |
| VEGFA | vascular endothelial growth factor A | Extracellular Space | growth factor |
| CSF1R | colony stimulating factor 1 receptor | Plasma Membrane | kinase |
| FGFR1 | fibroblast growth factor receptor 1 | Plasma Membrane | kinase |
| FYN | FYN oncogene related to SRC, FGR, YES | Plasma Membrane | kinase |
| LCK | lymphocyte-specific protein tyrosine kinase | Cytoplasm | kinase |
| MAPK15 | mitogen-activated protein kinase 15 | Cytoplasm | kinase |
| RET | ret proto-oncogene | Plasma Membrane | kinase |
| PPARD | peroxisome proliferator-activated receptor delta | Nucleus | ligand-dependent nuclear receptor |
| CD44 | CD44 molecule (Indian blood group) | Plasma Membrane | other |
| COL18A1 | collagen, type XVIII, alpha 1 | Extracellular Space | other |
| COL1A1 | collagen, type I, alpha 1 | Extracellular Space | other |
| ERRFI1 | ERBB receptor feedback inhibitor 1 | Cytoplasm | other |
| NF2 | neurofibromin 2 (merlin) | Plasma Membrane | other |
| SERPINE1 | serpin peptidase inhibitor, clade E, member 1 | Extracellular Space | other |
| MMP1 | matrix metallopeptidase 1 (interstitial collagenase) | Extracellular Space | peptidase |
| CTNNB1 | catenin (cadherin-associated protein), beta 1, 88kDa | Nucleus | transcription regulator |
| MYC | v-myc myelocytomatosis viral oncogene homolog (avian) | Nucleus | transcription regulator |
| SLC3A2 | solute carrier family 3 member 2 | Plasma Membrane | transporter |
|  |  |  |  |
| **SW837: down-regulated genes** | |  |  |
| CCL20 | chemokine (C-C motif) ligand 20 | Extracellular Space | cytokine |
| FLT3LG | fms-related tyrosine kinase 3 ligand | Extracellular Space | cytokine |
| TNFSF10 | tumor necrosis factor (ligand) superfamily, member 10 | Extracellular Space | cytokine |
| BRAF | v-raf murine sarcoma viral oncogene homolog B1 | Cytoplasm | enzyme |
| CDC42 | cell division cycle 42 (GTP binding protein, 25kDa) | Cytoplasm | enzyme |
| GCNT1 | glucosaminyl (N-acetyl) transferase 1, core 2 | Cytoplasm | enzyme |
| GNA12 | guanine nucleotide binding protein (G protein) alpha 12 | Plasma Membrane | enzyme |
| GNA13 | guanine nucleotide binding protein (G protein), alpha 13 | Plasma Membrane | enzyme |
| GNAS | GNAS complex locus | Plasma Membrane | enzyme |
| HPSE | heparanase | Plasma Membrane | enzyme |
| PLCG1 | phospholipase C, gamma 1 | Cytoplasm | enzyme |
| RHOB | ras homolog gene family, member B | Cytoplasm | enzyme |
| F2R | coagulation factor II (thrombin) receptor | Plasma Membrane | G-protein coupled receptor |
| GRN | granulin | Extracellular Space | growth factor |
| KITLG | KIT ligand | Extracellular Space | growth factor |
| MDK | midkine (neurite growth-promoting factor 2) | Extracellular Space | growth factor |
| NGF | nerve growth factor (beta polypeptide) | Extracellular Space | growth factor |
| AKT1 | v-akt murine thymoma viral oncogene homolog 1 | Cytoplasm | kinase |
| CSNK2A1 | casein kinase 2, alpha 1 polypeptide | Cytoplasm | kinase |
| ERBB3 | v-erb-b2 erythroblastic leukemia viral oncogene homolog 3 (avian) | Plasma Membrane | kinase |
| ERBB4 | v-erb-a erythroblastic leukemia viral oncogene homolog 4 (avian) | Plasma Membrane | kinase |
| KIT | v-kit Hardy-Zuckerman 4 feline sarcoma viral oncogene homolog | Plasma Membrane | kinase |
| MAP2K1 | mitogen-activated protein kinase kinase 1 | Cytoplasm | kinase |
| MAP2K4 | mitogen-activated protein kinase kinase 4 | Cytoplasm | kinase |
| MAPK1 | mitogen-activated protein kinase 1 | Cytoplasm | kinase |
| MAPK11 | mitogen-activated protein kinase 11 | Cytoplasm | kinase |
| MAPK7 | mitogen-activated protein kinase 7 | Cytoplasm | kinase |
| MAPKAPK2 | mitogen-activated protein kinase-activated protein kinase 2 | Nucleus | kinase |
| MST1R | macrophage stimulating 1 receptor | Plasma Membrane | kinase |
| MYLK | myosin light chain kinase | Cytoplasm | kinase |
| PDPK1 | 3-phosphoinositide dependent protein kinase-1 | Cytoplasm | kinase |
| PIK3CA | phosphoinositide-3-kinase, catalytic, alpha polypeptide | Cytoplasm | kinase |
| PIK3CB | phosphoinositide-3-kinase, catalytic, beta polypeptide | Cytoplasm | kinase |
| PRKCA | protein kinase C, alpha | Cytoplasm | kinase |
| PTK2B | PTK2B protein tyrosine kinase 2 beta | Cytoplasm | kinase |
| SYK | spleen tyrosine kinase | Cytoplasm | kinase |
| PPARG | peroxisome proliferator-activated receptor gamma | Nucleus | ligand-dependent nuclear receptor |
| ARRB1 | arrestin, beta 1 | Cytoplasm | other |
| CD24 | CD24 molecule | Plasma Membrane | other |
| EDN1 | endothelin 1 | Extracellular Space | other |
| FBLN1 | fibulin 1 | Extracellular Space | other |
| GAST | gastrin | Extracellular Space | other |
| HSP90AA2 | heat shock protein 90kDa alpha (cytosolic), class A member 2 | Unknown | other |
| IGFBP3 | insulin-like growth factor binding protein 3 | Extracellular Space | other |
| MUC1 | mucin 1, cell surface associated | Plasma Membrane | other |
| STMN1 | stathmin 1/oncoprotein 18 | Cytoplasm | other |
| TNC | tenascin C | Extracellular Space | other |
| TSC1 | tuberous sclerosis 1 | Cytoplasm | other |
| ADAM10 | ADAM metallopeptidase domain 10 | Plasma Membrane | peptidase |
| CASP3 | caspase 3, apoptosis-related cysteine peptidase | Cytoplasm | peptidase |
| PTP4A3 | protein tyrosine phosphatase type IVA, member 3 | Plasma Membrane | phosphatase |
| PTPN1 | protein tyrosine phosphatase, non-receptor type 1 | Cytoplasm | phosphatase |
| PTPN6 | protein tyrosine phosphatase, non-receptor type 6 | Cytoplasm | phosphatase |
| ARNT | aryl hydrocarbon receptor nuclear translocator | Nucleus | transcription regulator |
| CBL | Cas-Br-M (murine) ecotropic retroviral transforming sequence | Nucleus | transcription regulator |
| HBP1 | HMG-box transcription factor 1 | Nucleus | transcription regulator |
| NCOA3 | nuclear receptor coactivator 3 | Nucleus | transcription regulator |
| RELA | v-rel reticuloendotheliosis viral oncogene homolog A (avian) | Nucleus | transcription regulator |
| STAT1 | signal transducer and activator of transcription 1, 91kDa | Nucleus | transcription regulator |
| IGF1R | insulin-like growth factor 1 receptor | Plasma Membrane | transmembrane receptor |
| ITGB4 | integrin, beta 4 | Plasma Membrane | transmembrane receptor |
| APOE | apolipoprotein E | Extracellular Space | transporter |
