# Supplementary figures and images for: JC Virus Mediates Invasion and Migration in Colorectal Metastasis
Source: PLoS One. 2009 Dec 3;4(12):e8146. doi: 10.1371/journal.pone.0008146 (PMC2781631; doi:10.1371/journal.pone.0008146)

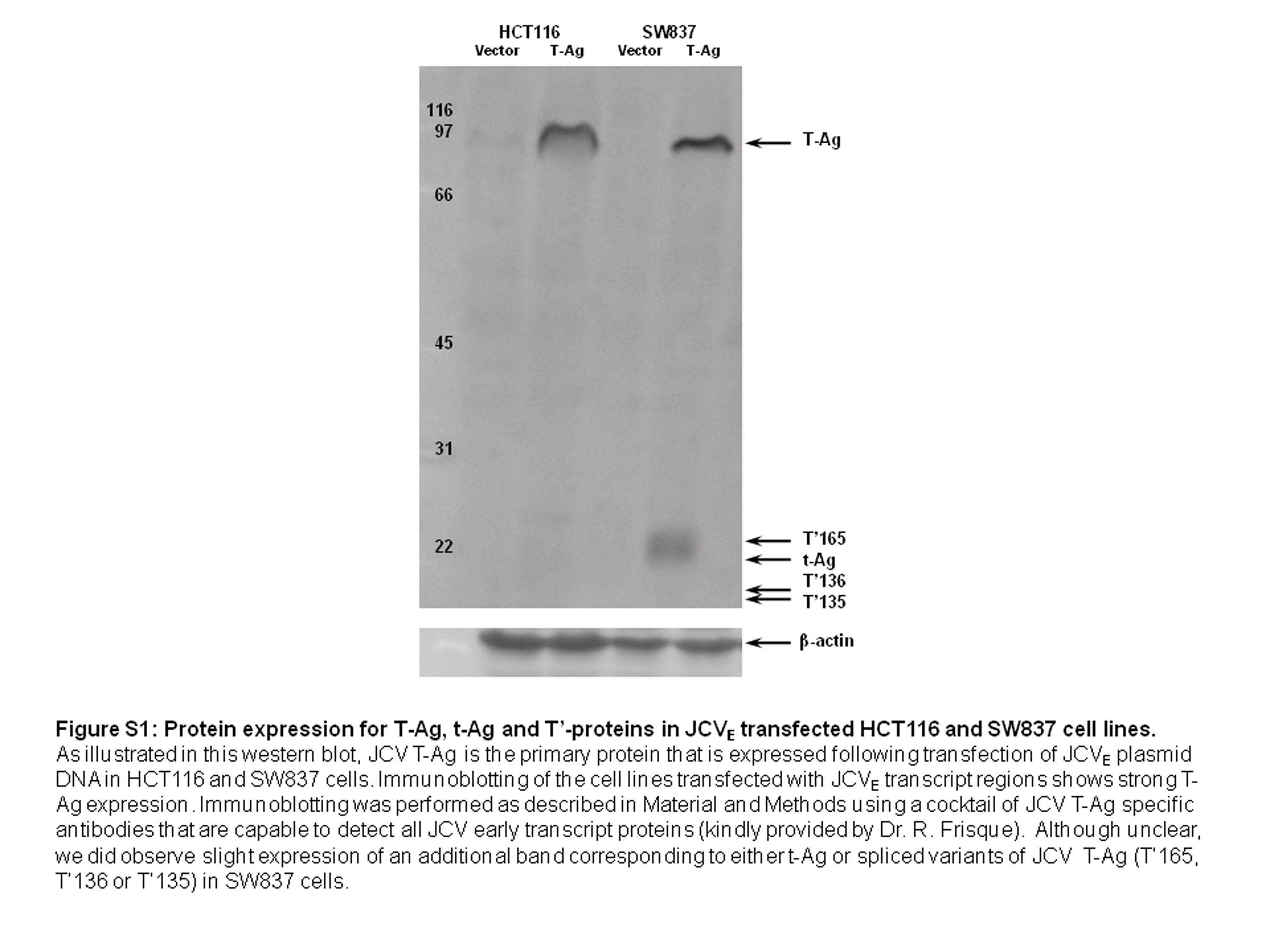

Supplement: Figure S1 — Protein expression for T-Ag, t-Ag and T'-proteins in JCVE transfected HCT116 and SW837 cell lines. (1.72 MB TIF) [file pone.0008146.s003.tif]
